# Mechanisms tagging senescent red blood cells for clearance in healthy humans

**DOI:** 10.3389/fphys.2013.00387

**Published:** 2013-12-25

**Authors:** Hans U. Lutz, Anna Bogdanova

**Affiliations:** ^1^Department of Biology, Institute of BiochemistryETH Zurich, Zurich, Switzerland; ^2^Vetsuisse Faculty, Zurich Center for Integrative Human Physiology (ZIHP), Institute of Veterinary Physiology, University of ZurichZurich, Switzerland

**Keywords:** human red blood cells, senescence, oxidative stress, hemoglobin, volume, vesicles, naturally occurring antibodies

## Abstract

This review focuses on the analysis and evaluation of the diverse senescence markers suggested to prime red blood cells (RBC) for clearance in humans. These tags develop in the course of biochemical and structural alterations accompanying RBC aging, as the decrease of activities of multiple enzymes, the gradual accumulation of oxidative damage, the loss of membrane in form of microvesicles, the redistribution of ions and alterations in cell volume, density, and deformability. The actual tags represent the penultimate galactosyl residues, revealed by desialylation of glycophorins, or the aggregates of the anion exchanger (band 3 protein) to which anti-galactose antibodies bind in the first and anti-band 3 naturally occurring antibodies (NAbs) in the second case. While anti-band 3 NAbs bind to the carbohydrate-free portion of band 3 aggregates in healthy humans, induced anti-lactoferrin antibodies bind to the carbohydrate-containing portion of band 3 and along with anti-band 3 NAbs may accelerated clearance of senescent RBC in patients with anti-neutrophil cytoplasmic antibodies (ANCA). Exoplasmically accessible phosphatidylserine (PS) and the alterations in the interplay between CD47 on RBC and its receptor on macrophages, signal regulatory protein alpha (SIRPalpha protein), were also reported to induce erythrocyte clearance. We discuss the relevance of each mechanism and analyze the strength of the data.

## Red blood cell ageing parameters and the criteria of evaluation

Over the years many investigators of red blood cells (RBC) and their biochemical properties have centrifuged whole blood and used the RBC pellet without actively removing leucocytes/platelets, despite their simple and selective depletion has been introduced and recommended almost 40 years ago by Beutler et al. (1976). Many investigators did not add protease inhibitors to the buffers in which RBC and their membranes/extracts were further processed. The omission of these precautions has been and still is the major reason for deviating data. Omission of leucocyte removal has dramatic effects on cell-age sensitive RBC properties, like e.g., hemolysis, echinocytosis, vesicle release, phosphatidylserine (PS) exposure, and band 3 protein clusterization as carefully studied by Antonelou et al. (2012). Thus, leucocyte-depletion renders transfusion of red blood cell units stored for 3–7 days safe (Hod et al., 2011) and ameliorates the effects of long stored blood (Phelan et al., 2010). Hence, the value of a set of data is highly dependent on having taken the first and where possible also the second precaution and we have selected the data for the present review accordingly. Other contradictions originate from a misnomer, for example when authors claim to illustrate a RBC property as a function of cell age, but compare properties of energy-starved RBC with those of freshly isolated ones (Girasole et al., 2012; Kim et al., 2012).

RBC undergo multiple changes while they age *in vivo*. Some of these remain hidden within RBC, others affect the properties of the cell directly, like the loss of cations and the loss of membrane with some hemoglobin by vesiculation that result in an increased cellular density. An increased density implies a higher cell age as has been established by the decreased activities of a number of intracellular enzymes, measured in the lysate (hexokinase, aldolase, pyruvate kinase, glutamate-oxalacetate transaminase) (Table 1). The decrease in activity of multiple enzymes is, however, not linear with cell age, but almost exponential from reticulocytes to mature cells [for a comprehensive review of these aspects, see reference Clark (1988)]. This disadvantage has forced many investigators to use as cell age parameters properties that change almost linearly with cell age, as exoplasmically located acetylcholinesterase (Cohen et al., 1976), the RBC creatine content (Fehr and Knob, 1979; Lutz and Fehr, 1979) and the ratio of two Coomassie-blue stainable bands (the 4.1a/4.1b ratio) (Mueller et al., 1987). This ratio illustrates the extent of deamidation of band 4.1b and luckily results in a change of the electrophoretic mobility of the protein in SDS polyacrylamide gels (Inaba and Maede, 1988). The separation of RBC according to their density has been achieved first by centrifuging RBC in an angle rotor where cells circulate to some extent in this highly viscous cell pellet (Murphy, 1973). With the availability of Stractan and Percoll it is the material that establishes a gradient or is arranged in a preformed gradient and allows RBC to better migrate to their actual density (Clark, 1985; Lutz et al., 1992). Aside of density separation aging RBC have been separated from each other by size, using counterflow centrifugation (elutriation), a method that offers a limited yield of separated cells and may best be combined with a preceding density centrifugation (Bosch et al., 1992).

**Table 1:** **Changes in RBC properties associated with aging**.

**RBC property or enzyme studied**	**Change of activity**	**Change of activity with cell age phase a/phase b**	**White cell removal**	**Species Studied**	**Data obtained by**	**References**
**METABOLIC ENZYMES**						
Lactate dehydrogenase/mg protein	–	Constant	Yes	Rabbit	*In vivo* biotinylation	Jindal et al., 1996
Phosphoglycerate kinase/mg protein	–	Constant	Yes	Rabbit	*In vivo* biotinylation	Jindal et al., 1996
Pyruvate kinase /mg protein	–	Constant	Yes	Rabbit	*In vivo* biotinylation	Jindal et al., 1996
Acid phosphatase/mg protein	–	Constant	Yes	Rabbit	*In vivo* biotinylation	Jindal et al., 1996
Pyruvate kinase/mg Hb	D	Exponential	Yes	Human	Density	Haram et al., 1991
Phosphofructokinase (PFK)/Cell number	D	Linear	Yes	Human	Density + Elutriation	Jansen et al., 1986
Glucose-6-phosphate dehydrogenase (G6PD)/cell number	D	Linear	Yes	Human	Density + Elutriation	Jansen et al., 1986
Hexokinase/cell number or mg/Hb	D	Exponential/linear	Yes	Human	Density + Elutriation	Haram et al., 1991; Piomelli and Seaman, 1993
**KINASES**						
Membrane associated casein kinase/mg protein	D	Linear	Yes	Rabbit	*In vivo* biotinylaiton	Jindal et al., 1996
Membrane casein kinase I/mg protein	D	Linear	Yes	Human	Density	Jindal et al., 1996
Casein kinase I/mg membrane protein	D	Linear	Yes	Rabbit	*In vivo* biotinylaiton	Jindal et al., 1996
Membrane protein kinase C (PKC)/mg protein	I	Increase/linear		Human	Density	Ramachandran and Abraham, 1989
Cytosolic protein kinase C (PKC)/mg protein	D	Exponential	Yes	Rabbit	*In vivo* biotinylation	Jindal et al., 1996
Cytosolic protein kinase C (PKC)/mg protein	D	Exponential	Yes	Human	Density	Jindal et al., 1996
Pyruvate kinase/cell number (PK) or mgHb	D	Exponential	Yes	Human	Density + Elutriation	Jansen et al., 1986; Piomelli and Seaman, 1993
Cytosolic CKII/mg protein	D	Exponential	Yes	Human	*In vivo* biotinylation	Jindal et al., 1996
Cytosolic PKA/mg protein	D	Exponential/constant	Yes	Human/Rabbit	Density	Jindal et al., 1996
**AMINO ACID MODIFICATIONS**						
Glutamate/oxalacetate transaminase/mg Hb	D	Exponential	Yes	Human	Density	Haram et al., 1991; Piomelli and Seaman, 1993
Aspartate amino transferase (ASAT)/cell number	D	Exponential	Yes	Human	Density + Elutriation	Jansen et al., 1986
AMP deaminase/mg Hb	D	Exponential/constant	Yes	Rabbit	*In vivo* biotinylation	Dale and Norenberg, 1989
**MARKERS OF SENESCENCE**						
Glutathione reductase (GR)/cell number	D	Linear	Yes	Human	Density + Elutriation	Jansen et al., 1986
HbA1c (glycated Hb) fraction/Whole Hb	I	Linear	–	Human	Biotinylation	Willekens et al., 2003; Cohen et al., 2008
Ratio of content of band 4.1a/4.1b/mg protein	D	Linear	Yes	Many	Density	Mueller et al., 1987; Inaba and Maede, 1988
Acetylcholinesterase units/mg Hb	D	Linear	Yes	Human	Density	Cohen et al., 1976
Creatine/cell number	D	Exponential	Yes	Human	Density	Fehr and Knob, 1979

*Changes in activity: I, increase and D, decrease; Phase a, reticulocytes mainly; phase b, mature RBC; Hb, hemoglobin; density, fractionation on the density gradient*.

## The changes occurring *in vivo*, when labeled RBC are re-introduced into the circulation

By far the most direct method to study *in vivo* aging of RBC is their biotinylation by N-hydroxysuccinimide-biotin and analyzing the properties of the biotinylated RBC during their life span in circulation by collecting the labeled RBC on avidin at various times after injection (Suzuki and Dale, 1987; Christian et al., 1993). The biotin derivative was dissolved in DMSO and a diluted sample was injected intravenously into dogs after bleeding to enhance the portion of young RBC in the labeled population (Christian et al., 1993). In humans bleeding was not an option and biotinylation had to occur *in vitro*. Hence, the fraction of labeled RBC was rather small toward the end of the *in vivo* survival study by 110–126 days (Franco et al., 2013). Nevertheless, it has been possible for the first time to demonstrate that all *in vivo* aged, biotinylated human RBC that were recovered 126 days post injection had increased amounts of membrane-bound IgG, but were not enriched at all in exoplasmically exposed PS (Franco et al., 2013). Similar findings have earlier been reported for dogs having a similar RBC survival time as humans. By 110 days biotinylated RBC carried 7 fold higher amounts of autologous IgG per RBC and massively increased amounts of membrane bound globin (Rettig et al., 1999). Unexpectedly, the density of biotinylated RBC increased primarily during the first 4 weeks of *in vivo* aging, but not or less thereafter as revealed by using preformed density gradients (Franco et al., 2013). Similar results were obtained earlier for biotinylated sickle RBC (Franco et al., 1998). The authors blame the density-separation technique for the unexpected results and suggest that density centrifugation should be combined with elutriation to achieve a better separation according to cell age. However, it cannot be excluded that *ex vivo* biotinylation of RBC in diluted DMSO and several washes had altered the properties of RBC that were not leucocyte-depleted. Nevertheless, analogous results on aging dog RBC confirm the unexpected finding. Dog RBC were *in vivo* biotinylated and revealed during survival signs of an accelerated aging in so far as a classical cell age parameter, the ratio of the band 4.1a/4.1b content had reached its maximum (full deamidation) in the biotinylated RBC portion long before the biotinylated RBC had reached their full survival time (Rettig et al., 1999). It may be possible that the findings were real, implying that a small fraction of aging RBC underwent a terminal density reversal by taking up sodium ions and water, as first described by Bookchin (Bookchin et al., 2000) and discussed in detail by Lew and Tiffert (2013). More studies are needed to clarify whether the terminal density reversal is induced by DMSO or the washes without white cell removal.

In the following chapters we address several parameters delineating age-related changes in healthy human RBC. Among them are oxidative stress, changes in cell volume and density, vesiculation, band 3 clustering, and binding of NAbs.

## The role of oxidative stress in red cell clearance

Cell aging is intimately related to the changes in the balance between production of pro-oxidants and their removal by anti-oxidative enzymes and scavengers to which reduced glutathione (GSH), NADH, NADPH, and ascorbate belong. Gradual accumulation of irreversibly oxidized and denatured proteins, in particular hemoglobin, occurs with ageing (Rifkind and Nagababu, 2013). Changes in activity of multiple enzymes, loss or reorganization of several proteins as well as alterations in plasma membrane lipid composition occur gradually in RBC over 120 days in circulation and are mainly caused by oxidative modifications. *De novo* synthesis of both proteins and lipids is absent in mature RBC. Accordingly, oxidized and denatured proteins accumulate in aging RBC and even more so because aging RBC lose free radical scavengers (Bartosz, 1981).

### Antioxidant capacity of RBC

**Figure 1:**
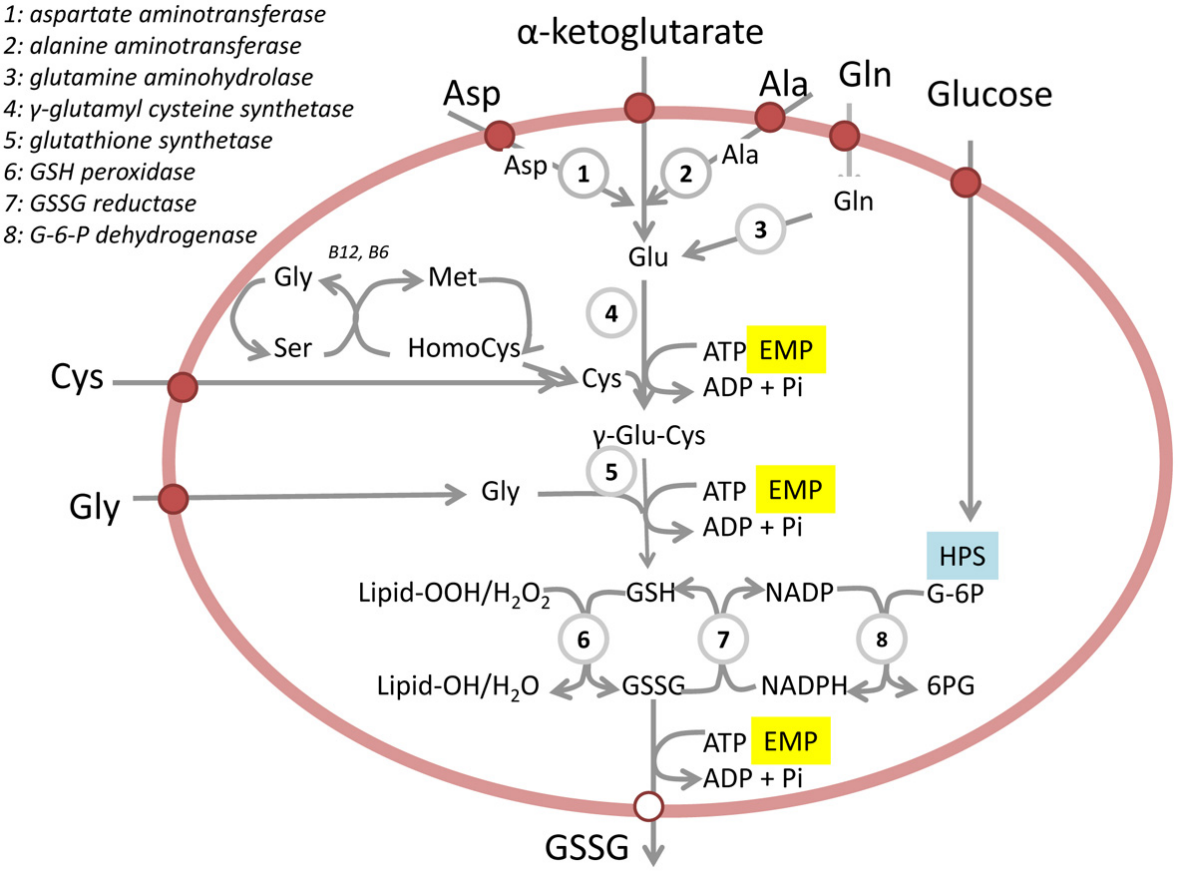
**Glutathione production and turnover in RBC**. Schematically presented are substrate delivery, glutathione synthesis, and glutathione handling in RBC. EMP stands for Embden-Meyerhof pathway (anaerobic glycolysis). HPS denotes hexose monophosphate shunt (pentose phosphate pathway). G-6P and 6 PG stand for glucose-6-phosphate and 6-phosphogluconolacton respectively. Glu, Gln, Gly, Cys, Ala, and Asp stand for glutamate, glutamine, glycine, cysteine, alanine, and aspartate respectively.

Among the antioxidants which prevent oxidation of protein thiols are GSH, NADH, and NADPH. Of these three compounds the half-cell redox potential based on the ratio of GSH to oxidized glutathione (GSSG) is postulated to be a reliable marker of the intracellular redox state (Schafer and Buettner, 2001). Reduced glutathione does not cross the plasma membrane passively and *de novo* synthesis of GSH is the only source of GSH in RBC. Facilitated unidirectional efflux of GSSG from the RBC is mediated by an ATP(GTP)-dependent transporter (RLIP76) and multidrug resistance protein 1 (MRP1) (Srivastava and Beutler, 1969; Bobrowska-Hagerstrand et al., 2001; Sharma et al., 2001). The ability of RBC to synthesize GSH, the presence of enzymes involved in GSH synthesis and GSSG formation (Figure 1) have been demonstrated in RBC lysates (Sass, 1968). Two substrates for glutathione synthesis, cysteine and glycine, are transported into the cells whereas glutamate is produced from aspartate and alanine by aspartate aminotransferase and alanine aminotransferase. As follows from the scheme in Figure 1 *de novo* synthesis of glutathione requires ATP and its reduction from GSSG to GSH requires NADPH.

The intracellular non-protein thiol levels, of which GSH is the major species, decrease substantially during the transformation of reticulocytes to mature RBC, but their concentration remains rather constant thereafter (Magnani et al., 1983; Piccinini et al., 1995). In all these studies the average intracellular GSH and GSSG concentrations were assessed without discriminating between the ratio of GSH to GSSG in the pre-membrane pool and that in the cytosolic core. Since the oxidative stress in aging RBC is particularly severe at the membrane, these measurements may not precisely reflect the aging-related shift in the redox state. The activity of γ-glutamylcysteine synthetase and that of GSH synthetase, two major enzymes involved in glutathione synthesis (Figure 1), remain unchanged in RBC with increasing density (Minnich et al., 1971) and get suppressed only in the cells showing the highest density on the Stractan gradient (Piccinini et al., 1995). The activities of key glycolytic enzymes hexokinase, pyruvate kinase, and glucose-6-phosphate dehydrogenase, as well as that of aspartate aminotransferase which provides glutamate for GSH synthesis, were the highest in the low density fraction and progressively decreased with an increase in red cell density [(Fornaini et al., 1985; Jansen et al., 1986) and Table 1].

Glucose transport across the RBC membrane decreases with cell age (Bosman and Kay, 1990). Young (least dense) RBC metabolize 2.5 times more glucose than old ones. On the other hand, the amount of glucose utilized via the hexose monophosphate shunt does not show any age dependence. Intracellular ATP levels drop by 30–40% following the down-regulation of Embden-Meyerhof (EMP) pathway (Cohen et al., 1976; Magnani et al., 1983), contributing to a shortage of GSH production in the densest RBC fraction.

RBC possess an efficient enzymatic machinery to process and detoxify reactive oxygen and nitrogen species, including superoxide dismutase (SOD1), catalase, peroxidases as well as glutathione peroxidase, peroxiredoxin 2 and glutaredoxin 1, which reverse oxidative thiol modifications on proteins and preserve enzyme activities from oxidative inactivation. The activities of some of these anti-oxidants, such as SOD1 and catalase decline during aging (Bartosz et al., 1978; Bartosz, 1980, 1981; Bartkowiak et al., 1983). Even extracellular SOD isozymes, including mitochondrial SOD2 (presumably from the endothelial cells), participate in detoxifying free radicals that cause RBC oxidation, as SOD2-deficient animals presented with higher rates of hemoglobin oxidation than controls (Mohanty et al., 2013). Further studies using knockout animals indicate that at least in rodents peroxiredoxin 2 and glutaredoxin 1 are the major enzymes detoxifying endogenous H_2_O_2_ in RBC (Lee et al., 2003; Johnson et al., 2010). Catalase on the contrary takes over detoxification of H_2_O_2_ produced externally (Johnson et al., 2010). RBC age-dependent changes in peroxiredoxin 2 and glutaredoxin 1 activities have not been studied yet.

### Sources of oxidative equivalents in senescent RBC

Cell age-related oxidation is largely a membrane-localized event, because free radical generators are compartmentalized. Most of them are localized within the membrane or are attached to membrane proteins from the cytosolic side, whereas enzymes detoxifying them as well as low molecular weight thiols are randomly distributed within the cytosol.

Auto-oxidation of hemoglobin is considered to be the major source of superoxide anion production in senescent RBC. Reduction of dioxygen to O^−^_2_ is associated with the generation of methemoglobin. Ferric iron of methemoglobin is then reduced to the ferrous state by hemoglobin reductase which uses NADH as a substrate. The methemoglobin concentration was shown to increase linearly with increasing RBC density (Imanishi et al., 1985; Rettig et al., 1999). *In vitro* studies of the hemoglobin auto-oxidation revealed that the reaction is slow (*k* = 0.0115 h^−1^) under normoxic conditions (Nagababu et al., 2002), but is facilitated dramatically upon partial deoxygenation (Abugo and Rifkind, 1994) and is maximal at pO_2_ of 1.33 kPa (Rifkind et al., 2004). Deoxyhemoglobin readily binds to the cytosolic domain of band 3 (Walder et al., 1984). This suggests that the levels of deoxyhemoglobin at the membrane surface exceed those in the cytosol. Furthermore, aging of RBC is associated with an increase in spectrin-hemoglobin complexes (Snyder et al., 1983) which contribute to an increased rigidigty of senescent RBC in dense fractions (Fortier et al., 1988). Accumulation of hemoglobin at the membrane surface and its auto-oxidation results in production of superoxide anion. Two further classes of enzymes which contribute to superoxide production are NADPH oxidases of which several isoforms are present in RBC (George et al., 2013) and endothelial NO synthase which is present in human and mouse RBC (Kleinbongard et al., 2006) and produces O_2_^−^ when L-arginine levels are low (Mihov et al., 2009).

Superoxide anion has a half-life of 10^−6^ s and undergoes a number of transformations depending on the availability of NO, superoxide dismutase (SOD), and H_2_O_2_. Interaction of O^−^_2_ with NO (*k* = 4–610^9^ M^−1^s^−1^) is four orders of magnitude faster than dismutation of O^−^_2_ to H_2_O_2_ catalyzed by SOD (*k* = 210^5^ M^−1^s^−1^). Thus, formation of peroxynitrite (ONOO^−^) from O^−^_2_dominates over the transformation of O^−^_2_ to H_2_O_2_ catalyzed by superoxide dismutase if NO is available (Borges-Alvarez et al., 2012). Peroxynitrite generated in this manner is believed to be a potent mediator of oxidative stress in RBC (Minetti et al., 2008; Rifkind and Nagababu, 2013). However, aging of RBC is not associated with an accumulation of nitrated tyrosine, the product of peroxynitrite reacting with membrane proteins or hemoglobin (Kikugawa et al., 2000). It is suggested that peroxynitrite production in circulating RBC is minimal due to the low abundance of deoxyhemoglobin (Winslow and Intaglietta, 2008). Thus, the importance of peroxynitrite as a mediator of oxidative stress in aging RBC remains questionable. NO may be viewed as a scavenger of superoxide radicals and therefore as a member of the antioxidative defense system. This defense system is challenged particularly under hypoxic conditions when partially oxygenated hemoglobin prone to auto-oxidation is formed. Deoxygenated hemoglobin has been shown to function as nitrite reductase transforming nitrite to NO being itself oxidized to methemoglobin (Gladwin and Kim-Shapiro, 2008).

Endogenous hydrogen peroxide formed by a SOD1-catalysed reaction as well as exogenous H_2_O_2_, diffusing into RBC from plasma, may be detoxified by catalase or peroxidase. However, when ferrous or ferric ions are available they catalyze reactions known as Haber-Weiss cycle in which hydroxyl radicals (HO) are formed. The hydroxyl radical has a half-life of 10^−9^ s and is extremely reactive and pro-oxidative. Iron ions are becoming accessible in the pre-membrane space during the process of oxidation and denaturation of membrane-bound hemoglobin occurring with aging of RBC (Low et al., 1985). Hemichrome accumulation and binding to the cytosolic domain of band 3 protein is a hallmark of RBC senescence.

### Targets of oxidants

RBC are one of the models of choice to monitor the effects of oxidants on proteins and lipids [e.g., Di Simplicio et al., 1998; Minetti et al., 2008]. However, relatively few studies refer to the monitoring of thiol modifications during ageing of RBC *in vivo* in healthy humans.

Hemoglobin, being the most abundant (98% of total protein content) protein in RBC, is the main generator of reactive oxygen species, the main target of oxidative damage and also a scavenger of free radicals. Detoxification of free radicals by hemoglobin is associated with the production of met-hemoglobin and oxidation of a single cysteine residue present in position 93 of the beta chain (Vitturi et al., 2013). The generated methemoglobin releases iron as Fe^+3^ in a chelatable form that can further propagate oxidative damage and induce binding of autologous IgG. Ferrali et al. synthesized an aromatic iron-chelator and applied it to mice in which oxidative damage and iron release were induced by a phenylhydrazine treatment. The RBC from animals treated with the chelator were protected from oxidative damage and from binding of autologous IgG (Ferrali et al., 2000).

Band 3 protein is also a target of oxidation, particularly when it has formed complexes with oxidized and denatured hemoglobin, hemichrome (Mannu et al., 1995). Accumulation of membrane-associated oxidized hemoglobin is considered to be one of the major (but not the only) trigger of band 3 cluster formation (Low et al., 1985; Schlüter and Drenckhahn, 1986). In addition, oxidation is a trigger for activation of src tyrosine kinases (Mallozzi et al., 2001) and inhibition of tyrosine phosphatases (Zipser et al., 2002), resulting in a cumulative tyrosine hyperphosphorylation of membrane target proteins. The src kinases including syk and lyn kinases phosphorylate tyrosines 8 and 21 of the cytosolic domain of the band 3 protein, thereby facilitating formation of high-molecular-mass band 3 aggregates (Pantaleo et al., 2009). Oxidation and poor glycosylation of band 3 further facilitates clustering of this protein (Pantaleo et al., 2009). Its tyrosine phosphorylation markedly reduces the affinity of band 3 to ankyrin, causing destabilization of the band 3-cytoskeleton interaction, increases the lateral mobility of band 3 within the membrane and induces vesiculation (Ferru et al., 2011). Both srk kinases syk and lyn and the tyrosine phosphatase PTP1B are redox-sensitive, be it because they possess cysteine residues in the kinase domains or indirectly, because their activity is controlled by the redox- and calcium-sensitive phosphorylation steps mediated in particular by protein kinase C alpha (Bordin et al., 2005; Knock and Ward, 2011).

## Changes in activity of ion transporter during RBC senescence

Transformation from reticulocytes to mature RBC and ageing of the latter is associated with radical re-organizations of the plasma membrane. Reduction in membrane surface by means of exocytosis during maturation of reticulocytes enables the cells to reduce or completely eliminate a number of receptors and ion transporters which play an active role in differentiation of erythroid precursor cells and are no longer required by mature RBC. Among the most well-known is the transferrin receptor. The number of copies per cell of Na,K-ATPase, Na/glycine transporter (Blostein and Grafova, 1987), nucleoside transporter, and K-Cl cotransporter (Canessa et al., 1987; Ellory et al., 1991) is reduced and renders the cell less energy-demanding and more stable.

Several ion transporters that are not extruded in exosomes undergo an age-dependent inactivation. The activity of the Gardos channel is reduced with ageing (Tiffert et al., 2007). Similarly, the number of copies of N-methyl D-aspartate (NMDA) receptors, non-selective cation channels mediating Ca^2+^ uptake into RBC, also decreases with cell age (Makhro et al., 2013). Plasma membrane Ca^2+^ pump was also reported to decline with cell age (Lucas et al., 1988; Samaja et al., 1989).

These conclusions may not necessarily apply to all cells within the light, medium or dense fractions of RBC, but to a majority of them. Recently extreme heterogeneity in responses of RBC to glutamate and homocysteic acid (Makhro et al., 2010, 2013), prostaglandin E2 (Kaestner et al., 2004), LPA (Wagner-Britz et al., 2013) has been recognized. This heterogeneity results from the inter-cellular variability in abundance of the corresponding ion transport pathways [NMDA receptors, voltage-gated Ca^2+^ channels (Cav2.1), and LPA receptors] in RBC of healthy humans (Makhro et al., 2013; Wagner-Britz et al., 2013).

In the following section the impact of vesicle release on RBC aging will be discussed. This process of membrane loss also contributes to the increase in density of senescent cells.

## Release of microvesicles and nanovesicles from aging RBC

It is known for many years that senescent RBC are smaller, denser than young cells and have lost membrane and hemoglobin. Nevertheless, many investigators have quantified N-acetylneuraminic acid (sialic acid) per young and per old RBC and then claimed that sialic acid decreased with cell age and the exposed remainder may act as a senescent cell marker, capable to trigger/mediate their selective removal (Gutowski et al., 1991; Bratosin et al., 1995). When the content of sialic acid was referred to a measure of the number of integral membrane proteins on young and old RBC, it was with ± 1.5% the same (Lutz and Fehr, 1979) and the cellular electrophoretic mobility remained unchanged during RBC aging (Luner et al., 1977). Despite the electrophoretic mobility remains unchanged, aging RBC loose a substantial portion of their membrane and content in form of microvesicles [reviewed in references Greenwalt (2006), Tissot et al. (2010)]. For a long time microvesicles were not in the focus of research, because those that are released *in vivo* are rapidly cleared and their relation to those from stored RBC was unclear. This situation has drastically changed since it has become obvious that the microvesicles from stored RBC have deleterious effects in transfused patients [for a review see Tissot et al. (2010)]. One of the deleterious effects comes from priming the respiratory burst of neutrophils (Jank and Salzer, 2011), which was, however, far more pronounced by microvesicles from RBC that were not leucocyte-depleted (Cardo et al., 2008).

The shedding of microvesicles from *in vivo* aging RBC was first illustrated by Dumaswala and Greenwalt (1984). By that time two types of *in vitro* RBC vesiculations had already been discovered as laboratory phenomena: Allan and coworkers had characterized vesicles released from RBC incubated with Ca^2+^ and ionophore (Allan and Michell, 1977) and Lutz and coworkers had studied vesicles released from ATP-depleted RBC (Lutz et al., 1977). Both types of vesicles contain hemoglobin and their membranes are enriched about 2-fold in exoplasmic acetylcholinesterase, lack spectrin and ankyin, but retain the integral membrane proteins band 3 and glycophorin to about 50%. Generation of microvesicles from stored RBC was also described in the 70s for storage in ACD (acid-citrate-dextrose) by Rumsby et al. (1977). These authors noted that stored RBC release hemoglobin-filled microvesicles that are depleted of spectrin, but contain integral membrane proteins. More recent data on the microvesicle release from stored and from Ca^2+^-loaded RBC that were leucocyte-depleted, have yielded the following insights. At the onset of RBC storage the released microvesicles differ in composition from those released from Ca^2+^-loaded RBC. After prolonged storage (beyond 21 days), when the ATP-content of stored RBC decreases rapidly, the total amount of released vesicular proteins increases exponentially, reaching 10 times the amount by 50 days as compared to 14 days of storage and their composition becomes comparable to that of Ca^2+^-induced vesicles (Salzer et al., 2008). It is possible that this late change in amount and properties of microvesicles from stored RBC may be the major reason for why long stored blood units were primarily responsible for the deleterious effects in transfused patients.

RBC shrinkage and echinocytosis precede the release of microvesicles. It is known that budding and the release of microvesicles from RBC incubated with Ca^2+^ and ionophore are induced by the formation of diacylglycerol (Allan and Michell, 1977). Microvesicle generation from ATP-depleted RBC also correlates with the breakdown of polyphosphoinositides to diacylglycerol on the inner monolayer (Müller et al., 1981). The diffusible diacylglycerol partitions into the outer monolayer and thereby contributes to membrane budding. Nevertheless, membrane budding and shedding of microvesicles yet require other changes, because 10 mM EDTA inhibited microvesicle release by 75% without affecting diacylglycerol production (Müller et al., 1981). Indeed, membrane budding is further dependent on exoplasmic exposure of phosphatidylserine (PS), a negatively charged phospholipid that normally is exclusively localized on the inner monolayer. Its exposure on the outer monolayer depends on activation of the scramblase by intracellular Ca^2+^, which is induced by ionophore (Nguyen et al., 2011) or by lysophosphatidic acid during RBC storage, but less upon prestorage leukodepletion (Nagura et al., 2013). It was further noted that prolonged storage of leucocyte-depleted RBC increases the susceptibility of RBC to stress-induced loss of phospholipid asymmetry. The effect was most pronounced for old RBC when experimentally evoked by a hyperosmotic shock, such that PS exposure increased 10-fold by 4 weeks storage and more so in old RBC (Bosman et al., 2011). Strong arguments in favor of PS exposure being required for microvesicle release come from (1) a bleeding disorder, the Scott syndrome, where RBC neither translocate PS across the membrane nor release microvesicles (Bevers et al., 1992) and (2) the finding that a specific inhibitor of the scramblase (R5421) inhibits both PS exposure and microvesicle release to more than 50% (Gonzalez et al., 2009).

The regions on RBC that become enriched in diacylglycerol and PS are associated with lipid rafts. Lipid rafts are microdomains in the plasmamembrane, rich in cholesterol and glycosphingolipids and contain unique proteins that differ in the two types of rafts known to exist in RBC. One type contains primarily stomatin and the flotillins (flotillin1 and 2). While the palmitoylated, hydrophobic stomatin is an integral membrane protein, the flotillins are peripheral membrane proteins with hydrophobic domains capable of associating with the bilayer (Salzer et al., 2002, 2008). Microvesicles (160 nm diameter) from RBC stored for more than 20 days (Kriebardis et al., 2008) and from Ca^2+^ loaded RBC (Salzer et al., 2002) contain band 3, are enriched in acetylcholinesterase and in stomatin (2-fold as compared to the original membrane), but threefold depleted of flotillin 2 that remains in the residual RBC membrane (Salzer et al., 2008). The thrombogenic activity of microvesicles released from stored RBC or from Ca^2+^ and ionophore treated RBC is comparable (Salzer et al., 2008). Microvesicles from long stored RBC contain heavily aggregated hemoglobin, band 3 aggregates and increasing amounts of autologous IgG (Kriebardis et al., 2008), while the residual RBC reveal a decrease in band 3 content, but yet a substantial increase in bound IgG (Kriebardis et al., 2007). Both types of microvesicles also contain small amounts of synexin and sorcin. The two peripheral membrane proteins, synexin and sorcin are predominantly associated with the second type of lipid raft in RBC and become the major proteins in the Ca^2+^-induced nanovesicles (60 nm diameter) (Salzer et al., 2002). Nanovesicles lack band 3, contain little stomatin, but are highly enriched in the two peripheral proteins synexin and sorcin, two proteins that were not known to exist in RBC (Salzer et al., 2002). Synexin is an annexin-like protein and binds in a Ca^2+^ dependent manner to sorcin and the cytoplasmic side of the budding membrane of nanovesicles. In other cells the two proteins play important roles in fusion of lamellar bodies (Sen et al., 1997).

The sequence of events in microvesicle and nanovesicle formation and release illustrates that cellular aging as evident from increasing amounts of oxidized hemoglobin, aggregated band 3 protein and surface-bound IgG appears to induce an entrapment of these irreversible endproducts in lipid raft-containing microvesicles that are rapidly cleared. The rapid clearance of these microvesicles occurs most likely by Kupfer cells and other macrophages via recognition of exposed PS, as established in an animal model using rats (Willekens et al., 2005). The selective release of vesicles containing aggregated band 3 with bound naturally occurring antibodies evidently prevents a premature recognition of the aging RBC by phagocytes.

## Naturally occurring anti-band 3 antibodies and complement in clearance of senescent human RBC

When Kay published her first paper on the “mechanism of removal of senescent cells by human macrophages” in 1975 it was the first contribution that addressed the selective clearance of *in vivo* aged human RBC (Kay, 1975). Senescent but not young RBC, as obtained by density separation, are phagocytosed by macrophages, while phagocytosis of *in vitro* aged RBC requires opsonization with autologous IgG. IgG eluted from senescent RBC induces phagocytosis of *in vitro* stored young RBC (Kay, 1978). These findings demonstrated that “opsonization of aging RBC by autologous IgG” represents the effect of “beneficial autoantibodies,” of “physiologic autoantibodies” that were also called natural antibodies or naturally occurring (auto)antibodies (NAbs). NAbs with beneficial roles had already been observed in 1942 (Kidd and Friedewald, 1942), but went almost unnoticed during the rapid development of classical immunology in the 60 and 70 s. This situation changed in the early 80 s with the description of NAbs against nine common human antigens (Guilbert et al., 1982) and the availability of the immunoblotting technique to visualize binding of whole IgG to individual membrane proteins (Towbin et al., 1979) and to embark on the affinity purification of NAbs to RBC membrane proteins like spectrin (Lutz and Wipf, 1982) and band 3 (Lutz et al., 1984).

In trying to identify the antigen exposed on aged RBC Kay carried out affinity purification of RBC proteins on immobilized IgG from senescent RBC and reported that this IgG NAb binds to a 62 kDa protein of old RBC (Kay, 1981). At the same time Lutz had evidence for band 3 oligomers to represent the antigen to which autologous IgG binds to (Lutz, 1981). The immune precipitate obtained with second antibody from detergent extracts of ^125^I-iodinated RBC contained material at 100 and 200 kDa and shared iodinated peptide maps with that of band 3 protein. Analogous precipitates from chymotrypsin-treated RBC yielded primarily a labeled 65 kDa fragment, implying that the intact antigen was band 3 (the two chymotryptic fragments of band 3 were ascribed the MW of 65 and 38 kDa to the carbohydrate-containing fragment, later with sequence data available these fragments were named 55 and 35 kDa fragment). Lutz and coworkers could establish that the antigenic site preexists in band 3 protein, but becomes accessible for NAb binding upon band 3 clusterization e.g., on spectrin-free vesicles in which band 3 is laterally mobile. This overview was concluded by suggesting that also in Kay's experiments the RBC-specific NAb may have bound to band 3/band 3 aggregates, but ended up in a 62 kDa fragment because of proteolysis (Lutz, 1981). The above mentioned data could not be published in a prestigious journal, because the existence of anti-band 3 NAbs had not yet been proven, though immunoblots with whole IgG revealed not only binding to both spectrin band 1 and 2 but also to band 3 (Lutz and Wipf, 1982). Eventually the entire information was published in pieces: Binding of autologous IgG was 14 times higher to spectrin-free vesicles than to ATP-maintaining RBC (Müller and Lutz, 1983), implying that NAb binding increases with oligomerization of integral membrane proteins. A detailed study of the glycoprotein topology on human RBC after an extensive aminogroup supplementation further showed that the 10^6^ band 3 molecules exist either as monomers or non-crosslinkable dimers of which the cross-linkable portion is minute, but increases at a confidence level of 0.06 by 0.4% from young to senescent cells (Schweizer et al., 1982). By leucocyte-depletion of RBC, by inhibiting proteases with phenylmethyl sulfonyl chloride (PMSF), by surface-^125^I -iodinating young and old RBC, by extraction with Triton and addition of a second antibody it was demonstrated that preexisting immune complexes contained band 3 monomers and oligomers in samples from old but not young RBC (Lutz and Stringaro-Wipf, 1983). Furthermore, preincubation of extracts from young RBC with autologous IgG resulted in immunoprecipitation of band 3, again suggesting the preexistence of antigenic sites in young RBC. Finally, anti-band 3 NAbs were purified from IgG of individuals and pooled IgG (Sandoglobulin) and characterized (Lutz et al., 1984). The antigenic site of anti-band 3 NAbs is located within the 65 kDa, but not the 38 kDa chymotryptic fragment of band 3 and antigenic band 3 protein is equally present on young and old RBC, implying that exposure requires an altered accessibility rather than an enzymatic generation of antigenic sites as suggested by Kay (Kay and Goodman, 1984). Indeed binding of purified anti-band 3 NAbs was about 10 times higher to band 3 oligomers than to monomers (Lutz et al., 1984).

In 1983 Kay followed the initial observation of Lutz et al. and reported that the senescent cell antigen is immunologically related to band 3, because not only the 62 kDa protein, but also intact band 3 inhibited phagocytosis of RBC incubated with IgG eluted from senescent RBC (Kay et al., 1983). In contrast to earlier work from her group, RBC studied in this paper were leucocyte-depleted and proteases inhibited in extracts. Correspondingly the stained band pattern of RBC ghosts did not reveal the 62 kDa fragment [for comparison see reference Kay (1981)]. The anti-senescent cell IgG was said to bind to both chymotryptic fragments from band 3, though the blot showed almost exclusive binding to the larger chymotryptic fragment (Kay, 1984). While this disagreement on anti-band 3 specificity continued (Kay et al., 1988; Kay, 1992), others built on the findings of Lutz et al. and extended their own studies on cell age dependent hemichrome binding to band 3 protein (Waugh and Low, 1985) by asking whether the clustering of band 3, which is induced by denatured hemoglobin might also promote binding of autologous IgG (Low et al., 1985). Indeed, oxidative damage induced by a phenylhydrazine treatment of RBC, known to result in hemichromes, greatly stimulated binding of autologous IgG and IgG binding colocalized with band 3 protein (Low et al., 1985). Sorette and Clark raised their doubts on the treatment with phenylhydrazine to simulate cell aging and showed that the highest IgG binding was to RBC with membrane lesions (Sorette and Clark, 1991). Drenckhahn a former collaborator of Low continued their joint studies and demonstrated that RBC of patients with unstable, oxidation-sensitive forms of hemoglobin (Heinz body anemia, hemoglobin Köln, and sickle cell anemia) revealed a co-clustering of denatured hemoglobin, of band 3 protein and of RBC-bound immunoglobulins, without having to pretreat RBC with an exogenous oxidizing agent (Schlüter and Drenckhahn, 1986). Low's group then isolated the densest human RBC and showed it contained 6 times more membrane bound hemichromes and 10 times more surface-bound autologous IgG than other RBC fractions of lower density (Kannan et al., 1991). They even managed to enrich the microscopic aggregates comprised of hemichromes, band 3 and spectrin, constituting 0.09% of the membrane protein, but carrying 55% of the total cell-bound IgG.

On a meeting in Israel Arese and Lutz got to know each other and decided to collaborate by using the phagocytosis assay with diamide-treated human RBC developed in Arese's lab (Bussolino et al., 1987) to investigate the functional properties of purified anti-band 3 NAbs. Anti-band 3 NAbs purified from pooled human IgG (Sandoglobulin) bound to SS group-containing band 3 oligomers and stimulated phagocytosis of diamide-treated RBC maximally at 10–20 μg/ml (Lutz et al., 1987). Largely unexpected was that efficient phagocytosis required C3b deposition, unless anti-band 3 NAbs were added at 20–100 times the physiological concentration. C3b binding to diamide-treated RBC was about two orders of magnitude higher than that of anti-band 3 NAbs and even occurred under alternative complement pathway conditions (Lutz et al., 1987). Likewise, senescent RBC having a five-fold lower creatine content than young RBC not only contained significantly more IgG, but also SDS-resistant complexes comprised of IgG and C3b as verified by immunoblotting with anti-IgG and anti-C3c (Lutz et al., 1988). Then, by quantifying the binding of labeled anti-band 3 NAbs to C3 it became obvious that anti-band 3 NAbs have a unique affinity for C3, while anti-spectrin NAbs not (Lutz et al., 1989). The weak affinity for C3 (2–3 × 10^5^ l/mol) at a site independent of the antigen binding domain is about 100 times higher than that of whole IgG, known to have a weak affinity for C3 (Lutz et al., 1993b). This affinity for C3 is responsible for the preferential formation of C3b-IgG and C3b_2_-IgG complexes during complement activation (Lutz et al., 1993c). Years later Jelezarova et al. verified that all such complexes contain dimeric C3b (C3b_2_-IgG), ester-bonded to one heavy chain of IgG (Jelezarova et al., 2003). It is the dimeric C3b within these complexes that renders them efficient activators of the alternative complement pathway. Thus, the ability of certain low affinity NAbs, like anti-band 3, to form such complexes during complement activation renders them far more efficient opsonins than judged from the number of bound antibody. Frank and collaborators had earlier found that certain induced IgG antibodies to bacteria also formed covalent complexes with C3b (C3b-IgG), which rendered these IgG molecules 3–4 fold more effective in stimulating complement deposition (Joiner et al., 1985).

To clarify the homeostatic role of purified anti-band 3 NAbs the survival of untreated and diamide-treated RBC was investigated in guinea pigs. In normal, but not in C3-deficient guinea pigs human anti-band 3 NAb binding significantly accelerated the clearance of diamide-treated guinea pig RBC (Giger et al., 1995). Likewise, a pretreatment of the animals with 200 μg of human band 3 protein slowed down the clearance of diamide-treated guinea pig RBC to the extent observed without anti-band 3 NAbs. In support of the role of complement Turrini et al. used either ZnCl_2_, acridine orange or melittin to cluster integral membrane proteins and determined binding of autologous IgG, complement C3c and quantified phagocytosis. The authors could confirm and extend the observations from the group of Lutz in so far as the clustering agent was only effective upon subsequent crosslinking of aggregated band 3 protein and only then induced binding of autologous IgG, deposition of C3c and phagocytosis (Turrini et al., 1991). IgG eluted following disaggregation of band 3 oligomers bound almost exclusively to band 3, its dimer and oligomers (Turrini et al., 1993). Disulfide-cross-linked band 3 dimers are indeed the minimal band 3 aggregates with enhanced affinity for anti-band 3 NAbs (Turrini et al., 1994). Similar conclusions were drawn by Beppu et al., using three different approaches to oxidize human RBC. All pretreatments increased binding of autologous IgG as well as of purified anti-band 3 NAbs and binding was inhibited by purified band 3 protein or by restoration of the protein SH groups (Beppu et al., 1990).

Oxidation or a treatment with a clustering agent and cross-linking of the generated clusters evidently simulates the prerequisites for clearance of RBC, but does not explain how band 3 clusters are formed during *in vivo* aging. Several authors have focused on the potential role of superoxide and NO, known to form peroxynitrite that induces lipid peroxidation and oxidizes hemoglobin to methemoglobin (Matarrese et al., 2005). Oxidative damage by peroxynitrite induces tyrosine phosphorylation of band 3 protein by several orders of magnitude and this phosphorylation enables the dissociation of band 3 from the spectrin-actin skeleton by lowering its affinity for ankyrin, whereby its cross-linkability and in plane diffusion are elevated (Ferru et al., 2011). Despite peroxynitrite can induce the oxidation of hemoglobin, can provide the prerequisite for band 3 protein oligomerization and can enable exoplasmic PS exposure, all these alterations are reversed by reactivation of glycolysis (Pietraforte et al., 2007). This implies that damage is minimal in aging RBC as long as ATP levels are maintained. Hence, peroxynitrite may contribute to oxidative damage in microvesicles, but not in aging RBC that maintain their ATP concentration at a high level and do not expose PS exoplasmically. An elegant recent report has finally brought the answer. Arashiki et al. have shown that efficient binding of methemoglobin to band 3 protein, requires a preceding peroxidation of the cytoplasmic portion of band 3 protein (Arashiki et al., 2013). Peroxidation of the cytoplasmic portion of band 3 protein results in carbonylation of this domain and this in turn enhances methemoglobin binding 5–7 fold. Bound methemoglobin then induces a conformational change which displaces ankyrin and gives rise to band 3 cluster formation.

## Anti-band 3 NAbs and induced anti-lactoferrin antibodies

Anti-band 3 NAbs, as isolated from plasma of healthy blood donors with blood group O or from Sandoglobulin bound to band 3 and exclusively to the 68/55 kDa chymotryptic fragment of band 3, but not to the carbohydrate-containing, 38/35 kDa fragment (Lutz et al., 1984). Eight years later Beppu reported that their anti-band 3 NAbs bind to the sialylated N-acetyllactosaminyl carbohydrate group localized within the 38 kDa fragment of band 3 (Beppu et al., 1992; Ando et al., 1994). Lutz et al. reinvestigated the specificity of anti-band 3 NAbs purified from pooled human IgG as found in Sandoglobulin. Anti-band 3 NAbs as prepared originally bound to band 3 and weakly to the cytoskeletal proteins band 4.2, 5 and spectrin. In the reinvestigation anti-band 3 NAbs were further purified by absorption on heat aggregated human IgG to deplete of anti-idiotypes. Anti-band 3 NAbs obtained in this manner bound on blots exclusively to band 3 and to the 55 kDa chymotryptic fragment of band 3, but not the 38 kDa chymotryptic fragment of band 3 (Lutz et al., 1993a). A detailed analysis further showed that binding of anti-band 3 NAbs to blotted band 3 protein from RBC membranes was neither inhibited by pretreating RBC with neuraminidase nor endo-β-galactosidase. In addition, its binding to band 3 and to the 55 kDa fragment of band 3 was not inhibited at all by 10 μg/ml lactoferrin (Lutz et al., 1993a). This was in complete contrast to the properties of Beppu's “anti-band 3 antibodies,” for which these authors showed an 80% inhibition of binding to oxidatively stressed RBC by 10 μg/ml lactoferrin (Beppu et al., 1992). The consequence was that many investigators in the RBC field considered the specificity of anti-band 3 NAbs as unresolved, as remaining controversial.

The second round of studying anti-band 3 specificity confirmed the initial characterization of anti-band 3 NAb preparations of Lutz and suggested that Beppu's group must have used a different type of starting material that upon purification on immobilized band 3 protein yielded anti-N-acetyllactosaminyl-specific IgG and anti-band 3 NAbs. This possibility was later rendered even more likely, when Ando et al. (1996) showed that 70% of their anti-band 3 antibodies bound to lactoferrin and 30% to a non-glycosylated portion of band 3 protein. The source of their starting material was ill defined: was serum from persons with blood group AB, (Beppu et al., 1992), blood group B (Beppu et al., 1990), normal adults (Fujino et al., 2000) and was further treated for 30 min at 56°C, at least in the first paper (Beppu et al., 1990). Nevertheless, none of these starting materials could explain the copurification of the two types of antibodies on band 3 protein. Years later, with additional clinical information available, Lutz came up with the suggestion that Beppu's group must have used serum/plasma from patients with one of the many autoimmune diseases that are characterized by anti-neutrophil cytoplasmic antibodies (ANCA) (Lutz, 2012). IgG anti-lactoferrin is one of the induced autoantibodies in patients with rheumatoid arthritis (Kida et al., 2011), systemic lupus erythematosus (Caccavo et al., 2005), ulcerative colitis (Peen et al., 1993; Teegen et al., 2009), cholangitis (Muratori et al., 2001), and many other chronic diseases. In contrast to this, healthy humans have no IgG anti-lactoferrin at all, as verified by ELISA on 34 (Caccavo et al., 2005) and 36 (Chikazawa et al., 2000) serum samples and by immunoblotting (Nässberger et al., 1994). Thus, the purified IgG antibody preparation from Beppu's group should not have been named “anti-band 3 Nab.” Their preparation is evidently a mixture of NAbs with an induced autoantibody.

On the other hand, the functional properties that Beppu's group has described for this mixture of anti-band 3 and anti-lactoferrin reveal an unexpected implication, namely that not only anti-band 3 NAbs, but also anti-lactoferrin antibodies bind to oxidatively stressed and aging RBC and together increase opsonization with IgG and complement (Beppu et al., 1996; Ando et al., 1997). The consequence is that patients with induced IgG anti-lactoferrin antibodies may suffer from anemia as was found for example in 6 SLE patients having no other ANCA type antibody, but anti-lactoferrin (Manolova, 2003). In other studies anemia accompanying the presence of anti-lactoferrin antibodies was characterized on the basis of diminished hemoglobin concentrations, elevated erythrocyte sedimentation rates (ESR) and/or a higher red blood cell distribution width (RDW) as for example in rheumatoid arthritis and SLE (Chikazawa et al., 2000; Caccavo et al., 2005) and inflammatory bowel diseases (Song et al., 2012). In these anemias of chronic disease (Weiss and Goodnough, 2005) erythropoiesis is not increased in proportion to the enhanced RBC clearance. No one in the medical field has considered the possibility that extra opsonization of aging RBC by IgG anti-lactoferrin may result in accelerated removal of normal, aging RBC and eventually in anemia. The presence of anti-lactoferrin in IgG eluates from ageing RBC of such patients would provide the proof.

The anemia developing from the enhanced clearance of aging RBC by bound anti-band 3 and anti-lactoferrin could be cured by treatment with human or bovine lactoferrin per os. Lactoferrin would complex anti-lactoferrin and thereby prevent it from binding to the 38 kDa fragment of band 3 on aging RBC. Indeed, treatment with lactoferrin appears to stop anemia of chronic disease: for example RBC from New Zealand Black mice, constitutively suffering from an autoimmune type RBC clearance, had fewer numbers of Coombs-positive RBC upon treatment with bovine lactoferrin (Zimecki et al., 1995). In humans 200 mg/day of orally applied lactoferrin in combination with erythropoietin normalized the hemoglobin concentration in 75 cancer patients with anemia of chronic disease (Macciò et al., 2010). In fact, lactoferrin was as effective as injection of 125 mg ferric gluconate per week along with erythropoietin, but reduced serum ferritin significantly. Lactoferrin was also effective in treating pregnant women with iron deficiency anemia: Oral administration of lactoferrin (100 mg/twice a day for 30 days), that was iron-saturated to 30%, increased the total serum iron and hemoglobin concentrations to a greater extent than administration of ferrous sulphate (156 mg/day) in 60 anemic women (Paesano et al., 2009, 2010). These authors also found that the lactoferrin treatment lowered the serum concentration of IL6 from 32 to 12 pg/ml and therefore think that this mitigated inhibition of ferroportin 1 on macrophages and thereby enhanced iron export to blood for efficient erythropoiesis (Paesano et al., 2010). These thoughts call for clinical trials in which patients with anemia of chronic disease will be studied for IgG anti-lactoferrin antibodies in their serum and if positive, will eventually be treated with oral lactoferrin.

## Other clearance mechanisms

All the above mentioned findings differ completely from the old idea that desialylation is the trigger for removal of senescent RBC (Henrich and Aminoff, 1983; Bratosin et al., 1995). Fudenberg and coworkers addressed this question from a different viewpoint and asked whether heat-eluted IgG from senescent RBC has the same specificity as anti-Thomson-Friedenreich antibodies (anti-T), that are generated against neuraminidase-treated RBC glycoproteins. A pretreatment of RBC with anti-T antibodies did not alter the binding of heat-eluted IgG (Khansari et al., 1983). Thus, the two binding sites differ from each other and IgG from senescent RBC do not bind at all to desialylated sites.

“Eryptosis or suicidal erythrocyte death” as portrayed by Lang et al. (2008) should not be considered a senescence-like mechanism in healthy humans. Eryptosis is induced by several types of stresses, in particular by entry of Ca^2+^ ions followed by the loss of potassium ions, cell shrinkage, exposure of exoplasmic phosphatidylserine, and recognition by phagocytes. Eryptosis is not a senescence variant, because RBC *in vivo* aged for 110–126 days do not expose PS as illustrated in chapter 2 (Franco et al., 2013). In fact Lang and coworkers have recently studied eryptosis on young and old human RBC and confirmed that PS exposure was similarly negligible in young and old RBC, but increased upon induction of eryptosis more so in old than young RBC (Ghashghaeinia et al., 2012). Eryptosis may contribute to accelerated RBC clearance in a number of systemic diseases like hemolytic uremic syndrome and in sepsis (Lang et al., 2008).

There were reports on yet another mechanism of how senescent RBC may be tagged. Galili and coworkers described anti-αGal antibodies as representing natural antibodies existing in the plasma of all humans at high concentrations of up to 1% of total IgG (Galili et al., 1986). Most of bound IgG could be stripped off from old RBC by galactose and RBC stripped from bound IgG were phagocytosed upon addition of anti-αGal. Clark and collaborators reinvestigated the role of anti-αGal and found that in eluates from senescent RBC 9–39% of total IgG had anti-αGal and 5–18% anti-band 3 specificity (Clark and Sorette, 1991). Considering the fact that human plasma contains 100–1000 times more anti-αGal than anti-band 3 NAbs, the retention of anti-αGal antibodies was due to incomplete removal by washing. A year later Galili and coworkers no longer reported that anti-αGal could be involved in removal of aged human RBC, because human cells lack the α1,3Gal structure, while this epitope has been found on surface glycoconjugates of other mammalian cells and on many bacterial surfaces (Hamadeh et al., 1992). In fact, anti-αGal is most likely an induced antibody rather than a NAb.

After Lutz had isolated and characterized anti-spectrin NAbs from human IgG, Wiener et al. found significantly increased plasma concentrations of anti-spectrin NAbs in splenectomized patients with β-thalassemia major and in patients with sickle cell anemia (Wiener et al., 1986). Surprisingly, eluates from patients' RBC also revealed anti-spectrin NAbs. The authors concluded that binding of these NAbs to intact RBC may either be due to abnormally exposed spectrin or because of a cross-reaction. Irrespective of the reason for bound anti-spectrin NAbs, the increased number of bound IgG as such most likely had a stimulating effect on the clearance of these cells. Berti and collaborators later reported analogous findings for RBC from rats and hypertransfused rats, incubated with induced rat anti-spectrin antibodies (Giuliani et al., 2000). In trying to understand the phenomenon the group of Lutz reported that an exoplasmic cross-linking of band 3 protein on human RBC not only enhanced binding of anti-band 3, but also that of purified ^125^I-iodinated anti-spectrin NAbs 7–10 fold at 0°C in the presence of nearly physiological IgG and HSA concentrations (Hornig and Lutz, 2000). Binding of anti-spectrin NAbs was not competed by anti-band 3 NAbs and bound anti-spectrin NAbs even stimulated binding of anti-band 3 NAbs by 30%. While anti-spectrin NAbs bound at physiologic tonicity to band 3 or an associated protein by virtue of their inherent polyreactivity (Hornig and Lutz, 2000), their interaction with other components is increased at low ionic strength (Heegaard, 1993). The role of anti-spectrin NAbs is most likely to opsonize ghosts, generated from hemolysing RBC in circulation, as has been studied in detail (Salhany et al., 2001).

RBC contain a surface protein that protects RBC from being phagocytosed, CD47 (integrin-associated protein). When RBC in circulation bump into macrophages, CD47 actively prevents engulfment by binding to SIRPα on macrophages and this interaction provides a “do not eat me signal” [for a review see reference Matozaki et al. (2009)]. Recently, Burger et al. suggested that CD47 may act like a molecular switch from suppression to promotion of phagocytosis, when it may facilitate recognition and phagocytosis of aged RBC by macrophages (Burger et al., 2012). The authors think that RBC aging induces a conformational change in CD47 whereby it binds thrombospondin-1 and then interacts with SIRPα and induces phagocytosis of aged RBC. The evidence is weak, because the required concentration of the thrombospondin-1 peptide exceeded the thrombospondin-1 concentration in plasma by 10^3^–10^4^ (Lutz, 2013) and the phenomena had been studied on RBC that (a) were not actively leucocyte-depleted and (b) were oxidatively damaged by CuSO_4_ and ascorbic acid rather than *in vivo* aged.

## Concluding remarks

Covered in this review are the mechanisms of senescence of RBC in healthy humans. These mechanisms may differ for RBC of patients with hereditary anemias or polycytemia and those observed in RBC during storage. In these cases calcium uptake, PS exposure and abnormal alterations in cell volume, membrane architecture, as well as redox balance may play a much more important role. The presentation of senescent RBC from healthy humans to phagocytes is mediated by complement, but initiated by naturally occurring anti-band 3 antibodies that bind to aggregated band 3 protein. A very similar, additive effect may have induced anti-lactoferrin antibodies in autoimmune diseases characterized by ANCA. The induced anti-lactoferrin antibodies bind to the carbohydrate portion of the band 3 protein and appear to accelerate clearance of otherwise normal senescent RBC and may induce anemia.

### Conflict of interest statement

The authors declare that the research was conducted in the absence of any commercial or financial relationships that could be construed as a potential conflict of interest.
